# Subject Advantage in L1-English Learners’ Production of Chinese Relative Clauses

**DOI:** 10.1007/s10936-022-09865-9

**Published:** 2022-04-24

**Authors:** Nozomi Tanaka, Alessia Cherici

**Affiliations:** Department of East Asian Languages and Cultures, Indiana University, 355 North Eagleson Avenue, Global and International Studies Building 2050A, Bloomington, IN 47405 USA

**Keywords:** Relative clause, Elicited production, Asymmetry, Chinese as a foreign language

## Abstract

This study investigated whether L1-English Chinese learners show a subject preference in their oral production of Chinese relative clauses (RCs) and whether they show animacy effects. We conducted a picture-based elicited production experiment that compared subject and object RCs, varying the object animacy between animate and inanimate. The results from thirty learners showed more targetlike performance in subject RCs than in object RCs, both at group and individual levels, regardless of object animacy. Error analyses revealed that more object RCs were converted into subject RCs than vice versa. These results point toward a clear subject preference despite conflicted findings in previous research on RCs in Chinese as a foreign language. Animacy influenced subject and object RCs alike: both types were easier to produce when featuring an inanimate object. We suggested similarity-based interference or distribution-based effects to account for this finding.

**Supplementary Information:**

The online version contains supplementary material available at 10.1007/s10936-022-09865-9.

## Introduction

Learners’ development of relative clauses (RCs) has been a focus of second language (L2) research for decades. Previous studies, many on L2 English, have shown a subject preference in relative clause acquisition: subject relative clauses (SRCs) like (1a) are associated with earlier emergence and mastery, shorter latencies in comprehension, and higher accuracy in production, compared to object relative clauses (ORCs) like (1b).

**Table:** 

(1)	a	the composer_i_ [ who __i_ adored the musician]	
	b	the musician_i_ [ whom the composer adored __i_]	(Lin, 2015, p. 1)

This asymmetry is in accord with the noun phrase accessibility hierarchy (NPAH), an implicational hierarchy proposed by Keenan and Comrie (1977), based on typological observations of about fifty languages: subject > direct object > indirect object > object of preposition > genitive > object of comparison.

The NPAH postulates that if a language has a relativization strategy for a certain position on the hierarchy, it should also be able to relativize higher positions: All languages that have a relativizing strategy can relativize subjects, those that can relativize direct objects can also relativize subjects, and so on. Keenan and Comrie (1977) alluded to the psychological ease of relativization as the source of this generalization. Subsequent research has found the NPAH to hold in child language development, foreign language acquisition, and adult language processing. In second language acquisition (SLA), the NPAH has been suggested as a linguistic universal (e.g., Ellis, 2008; Gass et al., 2013; Lightbown & Spada, 2013). Studies on the acquisition of typologically diverse languages, however, have questioned the extent to which the subject preference is generalizable, particularly in languages with pre-nominal RCs (Shirai & Ozeki, 2007) such as Mandarin Chinese (hereafter Chinese).

This study reports on a subject preference observed in the oral production of Chinese RCs by L1-English learners. First, we show that a picture-based oral production task is an appropriate measure of learners’ knowledge of RCs and that it eliminates confounds associated with comprehension/reading tasks and written production tasks. Second, we test the role of animacy, an important factor often neglected in L2 RC literature. Third, we provide robust evidence for an SRC preference, confirmed through group- and individual-level analyses, which persisted regardless of animacy manipulation, in a language that has previously shown conflicting results.

In the following sections, we summarize previous literature on Chinese RCs, review methodological issues and the role of animacy, and explain the rationale for the study design. We then describe the method and results. We conclude by discussing broader implications.

## Chinese Relative Clauses

A (restrictive) relative clause helps narrow down the reference of the noun it modifies. In the previous examples in (1), the modified noun (i.e., head) corresponds to the missing embedded subject (1a) or object (1b) of the dependent clause. A gap is postulated in the position of the missing argument corresponding to the head. Chinese RCs feature gaps too, but they are prenominal. In (2), the RC precedes the relativizer *de*, followed by the head.

**Table:** 

(2)	a	Chinese SRC						
		[ __i_	àimù	yīnyuèjiā	de ]	zuòqǔjiā﻿_﻿i﻿_		
			adore	musician	rel	composer		
		‘the composer who adored the musician’						
	b	Chinese ORC						
		[ zuòqǔjiā	àimù	﻿__﻿i_	de ]	yīnyuèjiā_i_		
		composer	adore		rel	musician		
		‘the musician who the composer adored’				(Lin, 2015, p. 3; gloss modified)		

As mentioned previously, a subject preference in language acquisition and processing is widely reported: SRCs are generally acquired earlier, comprehended faster, and produced with higher accuracy than ORCs. In Chinese, however, studies on child language acquisition, adult language processing, and second language acquisition have reported both SRC and ORC preferences. Table 1 summarizes RC research in L2 Chinese (For summaries of research on native speakers’ RC preferences see Lau & Tanaka, 2021; Xiong et al., 2019; Xu et al., 2019.)

**Table 1:** Summary of Chinese RC studies with learners

Study	L1	Proficiency	Method	Animacy	Preference
Chen (1999)	English^a^ Japanese^b^	MLL: 2 years minimum; Instructional level: Intermediate	Acceptability judgment Word ordering task	Head animacy varied Head animacy varied	O > S, no L1 effects, no mention of animacy effects n.s., no L1 effects, no mention of animacy effects
Chen (2017)	English^a^	n/a	Self-paced reading	Controlled (all animate)	S > O
	Korean^b^				None
Chen (2019)	English^a^	n/a	Truth-value judgment	Controlled (all animate)	S > O
	Korean^b^				None
Cui (2013)	English^a^; French^a^; Spanish^a^	MLL: 3 years	Self-paced reading; offline questionnaire	No mention	S > O (RT), O > S (accuracy)
	Japanese^b^; Korean^b^				n.s. (RT), O > S (accuracy)
Dai (2010)	English^a^; Japanese^b^; Korean^b^ (others unspecified)	Intermediate-high minimum	Sentence combination	No mention	O > S (subject-modifying), S > O (object-modifying)
Li and Wu (2013)	English^a^	HSK 1.0: Advanced	Corpus analysis	No mention	S > O (all), n.s. (transitive only)
Li et al. (2016)	Italian^a^; Russian^a^; Thai^a^; Vietnamese^a^; Japanese^b^; Korean^b^ Mongolian^b^	HSK (version unclear): Intermediate minimum	Self-paced reading	No mention	S > O (accuracy, RT)
Packard (2008)	English^a^; German^a^; Indonesian^b^; Japanese^b^; Korean^b^	MLL: 8 years; Instructional level: 3rd and 4th year	Self-paced reading	Not controlled	O > S (RT), n.s. (accuracy)
Sung et al. (2016)	Japanese^b^	MLL: 2.1 years; CEFR: A1–B1	Eye-tracking (reading)	No mention	O > S (RT), n.s. (accuracy)
Xu (2013)	English^a^	MLL: 5.5 months; Instructional level: 2nd semester	Listening comprehension	Animate subject; object animacy varied	S > O (animate S/O, no determiner), inanimate > animate object
Xu (2014a)	English^a^	ILR scale: 2 (Limited Working Proficiency) minimum	Sentence combination	No mention	n.s. (accuracy), S > O (error types)
Xu (2014b)	English^a^	ILR scale: 2 or 2 +	Timed reading + grammaticality judgment	Controlled (all animate)	S > O (RC preceding determiner/classifier)
Yao (2018)	English^a^; French^a^; German^a^; Hebrew^a^; Italian^a^; Portuguese^a^; Russian^a^; Spanish^a^; Japanese^b^; Kazakh^b^; Korean^b^; Indonesian^b^	Instructional level: Advanced	Self-paced reading	Subject and object animacy varied	S > O (interpretation of ambiguous RCs), O > S (RT), inanimate-head > animate-head ORC

Animacy information is based on the explicit mention in the method section and/or the full list of stimuli when available. The symbol ‘>’ denotes a preference for the condition to the left. CEFR = Common European Framework of Reference for Languages; HSK = *Hanyu Shuiping Kaoshi* (Chinese Proficiency Test); ILR = Interagency Language Roundtable; MLL = mean length of learning; n.s. = no statistical significance; RT = reading time

^a^Postnominal RCs; ^b^Prenominal RCs

Previous research leaves two issues underexplored. The first issue applies specifically to Chinese (and other languages with prenominal RCs); because Chinese allows null arguments and flexible word order, prenominal RCs can be misanalyzed as part of the main clause until the parser reaches the relativizer *de* (Lin & Bever, 2011). Such main clause ambiguity creates a garden-path effect that can interfere with experimental results. L2 research on comprehension of Chinese RCs has not addressed this issue, but L1 studies that eliminated such confounds have found a clear subject preference (Jäger et al., 2015; Lin & Bever, 2011).^1^ Another way to avoid main clause ambiguity effects is to test production, and production studies involving native speakers (NSs) found a subject preference (Hsu et al., 2009; Lin, 2013). Xu (2014a), the only study reporting on L2 Chinese RC production to date, did not find a significant difference between SRCs and ORCs, although error patterns pointed toward a subject preference. However, Xu (2014a) employed a written task, which may have introduced additional difficulty associated with Chinese orthography. We used an oral production task instead, which allowed us to test whether there is a clear subject preference by eliminating main clause ambiguity and orthography-related difficulties.

The second issue concerning animacy effects applies to L2 studies on RC in general. Animacy is deemed to exert an important modulating effect on subject-object asymmetry: in general, ORCs were found to be more easily comprehended and produced with an inanimate head than with an animate head in L1 research (Kidd et al., 2007; Mak et al., 2002; Traxler et al., 2002, a.o.). However, the effects of animacy are an underexplored topic both in L2 production research and L2 Chinese research. To date, only Jeon and Kim (2007) and Ozeki and Shirai (2007) have investigated the role of animacy in L2 production. Previous studies on L2 Chinese, except Xu (2013), included only animate subjects and objects or did not control for animacy. As the first L2-Chinese RC production study to investigate animacy, this study contributes to the understanding of the role of animacy in L2 production.

## The Current Study

To fully explore RC asymmetry, we must study both L1 and L2 acquisition of languages typologically different from English and other European languages. Our study addresses the gaps in the literature by testing L2-Chinese learners using a picture-based oral production task that manipulates animacy. Our research questions are: (a) Do learners of Chinese show an SRC preference in production? (b) Does learners’ RC production show animacy effects?

## Rationale of the Design

In order to obviate the comprehension-related confounds and scarcity of production research, we employed a picture-based oral elicited production task, which has been successfully employed in research on child language (e.g., Hsu et al., 2009; Kim & O’Grady, 2016) and heritage language (Lee-Ellis, 2011). This paradigm enables pragmatically felicitous contexts (Hamburger & Crain, 1982), forcing the participants to build RCs from scratch (e.g., Kim & O’Grady, 2016). Previous L2 studies (including Xu, 2014a) commonly employed written production tasks (e.g., sentence combination, sentence completion), which are easy to include in classroom activities and yield more controlled production data, but they are metalinguistic in nature. Moreover, an oral task eliminates the additional challenge of orthography for learners of Chinese (e.g., Sung & Wu, 2011).^2^

Our production task includes both animate and inanimate objects in order to examine the possible influence of head animacy in ORCs. Xu’s (2013) L2-Chinese RC comprehension study found no asymmetry between SRCs and ORCs when subjects were animate and objects were inanimate. Mitsugi and Shirai (2017) showed no asymmetry for Japanese NSs, whereas L1-Korean learners had difficulty with ORCs regardless of animacy. Meanwhile, Ozeki and Shirai’s (2007) L2-Japanese study and Jeon and Kim’s (2007) L2-Korean study showed a strong association between animate heads and SRCs as well as between inanimate heads and ORCs in production. Our production data will provide further insight in this regard.

Two additional factors are relevant in SLA. The first is the role of L1. Some studies argue that learners’ L1, particularly word order and RC head position, influences how and whether an RC asymmetry is manifested (Chen, 2017, 2019; Cui, 2013; Kanno, 2007; Mitsugi & Shirai, 2017; O’Grady et al., 2003). The role of L1 was not the focus of our study and therefore we did not compare Chinese learners with different L1s; rather, we controlled for L1 background: All participants’ native language is English, so any variability in the data is not due to L1 differences.^3^ The second factor is the role of instruction. Intervention studies have found that the NPAH has pedagogical implications: Instruction on one type of RC, say ORCs, facilitates the learning of RCs in higher positions (e.g., SRCs), but not in lower positions of the NPAH (e.g., Ammar & Lightbown, 2005; Croteau, 1995; Eckman et al., 1988; Gass, 1982). This study does not address instructional effects.

## Method

### Participants

Thirty-seven learners were recruited from second- and third-year Chinese classes at a large American university.^4^ Seven were excluded during the analysis for not producing any RCs, leaving thirty learners (age of testing: 18‒60, mean 21.8, SD 8.3; age of L2 onset: 14‒57, mean 19.5, SD 8.4) for analysis. (Supplementary File 1 provides details.) In addition, we tested the materials with fifteen adult NSs of Chinese (mean age: 28.8 years) residing in the United States.

### Materials and Procedure

Participants were tested individually in a quiet room and completed all tasks in a single session lasting approximately 30 minutes: an RC production task, a picture narration task to assess proficiency, a verbal vocabulary check to ensure their familiarity with the words used in the RC task, and a language history questionnaire (Li et al., 2014). Participants received compensation.

#### RC Production Task

**Fig. 1:**
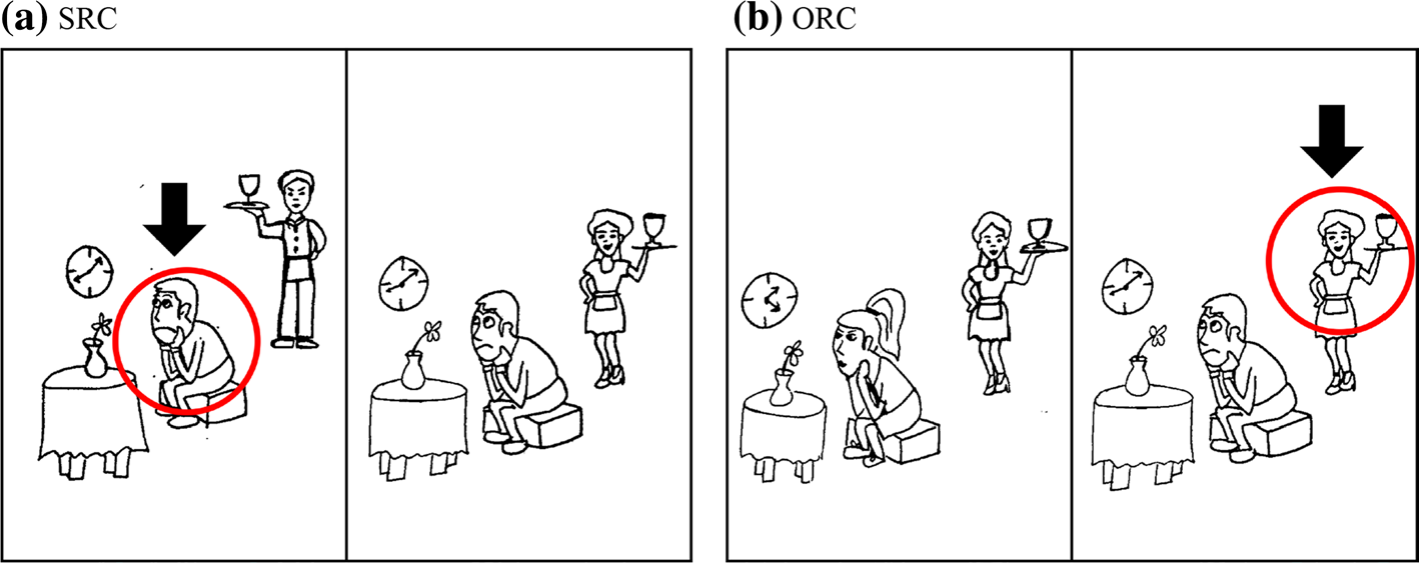
Sample items in the animate subject, animate object (A-A) condition

The RC production task followed a picture-based elicitation method used in previous studies (e.g., Hsu et al., 2009; Kim & O’Grady, 2016) with slight modifications. The stimuli consisted of twenty items, each containing a two-picture panel. Ten were intended to elicit SRCs, and ten to elicit ORCs. For each RC type, five items described events where both subject and object were animate (A–A condition), and five described events with an animate subject and an inanimate object (A–I condition). All lexical items used were from lessons that had been covered in the learners’ textbook (Liu et al., 2010). Participants’ limited vocabulary precluded the inclusion of items with inanimate subjects. Figure 1a shows an SRC item with two boys: one is waiting for a male server and the other is waiting for a female server. Figure 1b shows an ORC item, with two female servers: a girl is waiting for one, and a boy is waiting for the other. This design satisfies Hamburger and Crain’s (1982) felicity conditions postulating that the pragmatically felicitous context which motivates the use of an RC requires multiple referents of the same kind differing with respect to a specific characteristic.

In each trial, participants first heard a description introducing referents from left to right using declarative clauses, accompanied by a red circle in each picture to draw participants’ attention to the intended referent. The example in (3) describes the pictures in Fig. 1a.

**Table:** 

(3)	Zhè-ge	nánhái	zài	děng	nán-fúwùyuán.	Zhè-ge	nánhái	zài	děng	nǚ-fúwùyuán.
	this-cl	boy	asp	wait	male-server	this-cl	boy	asp	wait	female-server
	‘This boy is waiting for the male server. This boy is waiting for the female server.’									

Then, the red circle disappeared, and participants heard the question in (4).

**Table:** 

(4)	Jiàntóu	zhǐ-zhe	shéi/shénme?
	arrow	point-asp	who/what
	‘Who/what is the arrow pointing to?’		

An arrow, accompanied by a beep, appeared pointing to one of the entities in the pictures. Participants described the entity to which the arrow was pointing to an experimenter, who saw the same illustrations but not the arrow. Participants would have to produce a complete RC to differentiate the two referents of the same kind.

The stimuli were presented on a laptop computer using Microsoft PowerPoint, starting with the instructions (in the L1), and three practice items. The animacy conditions were presented in two blocks, with orders counterbalanced among participants.^5^ The order of stimuli within each block was pseudorandomized. Supplementary File 2 provides the complete list of test sentences.

The RC production task was audio recorded and later transcribed and coded for analysis. We considered as target responses well-formed RCs with all the necessary components: embedded verb, embedded NP, relativizer *de*, and head noun (5a). Mistakes in tone and minor lexical changes or errors (e.g., ‘doctor’ instead of ‘nurse’, ‘hit’ instead of ‘kick’) were disregarded. One type of nontargetlike response was passivization, which turned ORCs into SRCs. Head errors were RCs whose head was a wrong referent, whereas head and role reversal errors (5b) were utterances in which the head referent was wrong and its grammatical role was reversed.

**Table:** 

(5)	a	Target response (ORC)				
		[nánhái	(zài)	zuò	de]	dàngāo
		boy	asp	make	rel	cake
		‘the cake that the boy is making’				
	b	Head and role reversal error (SRC)				
		[(zài)	zuò	dàngāo	de]	nánhái
		asp	make	cake	rel	boy
		‘the boy who is making the cake’				

A fourth error type was failure to produce an RC (e.g., using declaratives, free-standing NPs, possessive NPs in lieu of RCs).

#### Picture Narration Task

The picture narration task (Hwang, 2020; Park, 2014; Song & Schwartz, 2009; Unsworth, 2005; Whong-Barr & Schwartz, 2002) consisted of three sets of four pictures, each portraying a sequence of everyday-life actions aiming to elicit (semi-)spontaneous data. Participants were asked to tell a story in Chinese for each set, providing as much detail as they wanted. This task type has several advantages over traditional proficiency measures, such as standardized proficiency tests (Hwang, 2020; Unsworth, 2005): It requires no reading or writing, which works well for learners of languages with difficult orthography; it involves storytelling, an activity familiar to most learners; it focuses on content/meaning, rather than form, thus being less metalinguistic or test-like, which alleviates performance-related stress; it is not time-consuming and can be easily integrated into the experimental procedure; and it does not require advanced vocabulary or complex syntax, making it appropriate for learners with varying proficiency.

Participants’ responses were transcribed and coded for T-unit boundaries and accuracy. We then counted number of T-units, number of error-free T-units, and number of verbs for each participant, based on which we calculated three subscores (Hwang, 2020). For the morphosyntactic complexity subscore, we used verbal density as the measurement, calculated as the number of verbs divided by the number of T-units. For the lexical complexity subscore, we used Moving-Average Type-Token Ratio (MATTR; Covington & McFall, 2010) following Hwang’s (2020) proposal to replace Guiraud’s index, which tends to get smaller as production lengthens and penalizes learners who produce more. MATTR was computed by calculating the average of the type-token ratio for a sequence of ten consecutive words, moving this ten-word window one word at a time until it reached the end of the transcribed narrative. For the morphosyntax and lexical accuracy subscore, we used error-free T-units, calculated as the number of error-free T-units divided by all T-units. The three subscores were converted into *z-*scores and combined into a final proficiency score, ranging from −3.88 to 3.54 (Supplementary File 1 provides further details).

## Results

This section reports the group-level and individual-level analyses to answer our research questions regarding whether L2-Chinese learners show (a) an SRC preference and (b) animacy effects in their production of RCs.

### Group Results

The RC production task was tested with fifteen NSs of Chinese to ensure it elicited intended responses. The NSs performed at ceiling with SRCs in both A–A and A–I conditions (90.67% and 100%, respectively). Their accuracy rates for ORCs were lower (A–A: 46.67%; A–I: 88.00%) due to a high rate of passivation (A–A: 42.67%; A–I: 6.67%), as already observed by Hsiao and MacDonald (2016) and Hsu et al. (2009). They rarely made errors (SRC: 2.67%; ORC: 6.67%) or failed to produce RCs (SRC: 1.33%; ORC: 5.33%), indicating that the stimuli were appropriate to elicit the intended responses.

As Table 2 documents, learners produced more targetlike SRCs than ORCs in both the A-A condition (SRC: 54.67%, ORC: 43.33%) and the A-I condition (SRC: 77.33%, ORC: 58.67%). Unlike the NSs, the learners rarely produced passives and frequently avoided producing RCs altogether.^6^ Head and role reversal errors were more frequent with (intended) ORCs than SRCs. This means that ORCs were converted into SRCs more frequently than vice versa, providing additional support for an SRC preference (Lee-Ellis, 2011; Xu, 2014a).

**Table 2:** Learners’ response types

		A-A	A-I	Overall
SRC	Targetlike	82 (54.67%)	116 (77.33%)	198 (66.00%)
	Head error	0 (0.00%)	1 (0.67%)	1 (0.33%)
	Head and role reversal error	3 (2.00%)	3 (2.00%)	6 (2.00%)
	Failure to produce RC	65 (43.33%)	30 (20.00%)	95 (31.67%)
Total		150	150	300
ORC	Targetlike	65 (43.33%)	88 (58.67%)	153 (51.00%)
	Passive	4 (2.67%)	5 (3.33%)	9 (3.33%)
	Head error	1 (0.67%)	0 (0.00%)	1 (0.33%)
	Head and role reversal error	6 (4.00%)	13 (8.67%)	19 (6.33%)
	Failure to produce RC	74 (49.33%)	44 (29.33%)	118 (39.33%)
Total		150	150	300

The data were fit into a binomial logistic mixed-effects model using the lme4 package (Bates et al., 2015) in R Version 4.1.1. The dependent variable was accuracy, with targetlike responses coded as 1 and all other responses as 0. The fixed effects included proficiency as a covariate (centered), sum-coded RC type (SRC vs. ORC), animacy (A–A vs. A–I), list (A–A first vs. A–I first) and all interactions among proficiency, RC type, and animacy. We included the maximal random effect structure that allowed the model to converge (Barr et al., 2013): participants and items as random intercepts, and by-participant effects of RC type and animacy as random slopes. The model reported converged without singular fit. As Table 3 shows, the main effects of RC type (*p* = 0.02), animacy (*p* = 0.01), and list (*p* = 0.03) were statistically significant. Considering that the intercept (0.38) represents the grand mean in sum-coded data, the model predicts higher accuracy for SRCs (0.38 + 0.54) than ORCs (0.38 − 0.54), higher accuracy for A–I items (0.38 + 0.94) than A–A items (0.38 − 0.94), and higher accuracy when the A–A block precedes the A–I block (0.38 + 1.36) than the opposite block order (0.39 − 1.36).^7^ The lack of interaction between RC type and animacy (*p* = 1.00) indicates an SRC preference regardless of animacy. The effect of proficiency was not significant (*p* = 0.09).

**Table 3:** Output of the binomial logistic mixed-effects model (coefficients in log-odd ratios)

	Estimate	Standard error	*z*-value	*p*-value	
Intercept	0.38	0.56	0.69	0.49	
Proficiency	0.56	0.33	1.69	0.09	
RC type (SRC vs. ORC)	0.54	0.24	2.25	0.02	*
Animacy (A-A vs. A-I)	− 0.94	0.37	− 2.55	0.01	*
List (A-A first vs. A-I first)	1.36	0.63	2.16	0.03	*
Proficiency × RC type	− 0.13	0.14	− 0.93	0.35	
Proficiency × Animacy	0.17	0.24	0.71	0.48	
RC type × Animacy	0.00	0.23	0.00	1.00	
Proficiency × RC type × Animacy	− 0.02	0.12	− 0.19	0.85	

Model: Accuracy ~ Proficiency * RC type * Animacy + List + (1 + RC type * Animacy | Participant) + (1 | item). *p < 0.05, **p < 0.01

### Individual Results

In RC asymmetry research, it is customary to report whether the group-level asymmetry is also observed at the individual level to provide a picture of individual differences. Supplementary File 1 reports the token number of target-like responses for each participant. The interpretation of such data, however, differs depending on the study. Lee-Ellis (2011), who used a similar elicited production task with heritage language learners of Korean, determined individual-level asymmetry by categorizing participants based on whether target responses in SRC and ORC differed for more than two tokens. It is unclear, however, how the two-token difference was justified statistically. For this reason, we set a threshold of success using binomial distribution (Volpato & Adani, 2009). Learners were considered successful when they produced at least nine out of ten targetlike responses for each RC type, a number required for above-chance performance according to the binomial distribution (chance level = 0.5, *p* < 0.05).^8^ We divided learners into four categories: (a) successful with both RC types (*n* = 4), (b) more successful with SRCs than ORCs (*n* = 7), (c) more successful with ORCs than SRCs (*n* = 2), and (d) successful with neither (*n* = 17). While most learners did not reach the threshold for success with either SRCs or ORCs, seven were more successful with SRCs and, crucially, only two were more successful with ORCs. Overall, 11 learners performed above chance on SRCs, while only six learners did so on ORCs. Individual performance, therefore, also indicates a subject preference.

## Discussion

This study investigated L1-English Chinese learners’ production of RCs using a picture-based oral elicited production task. The research questions we set out to answer were: (a) whether learners of Chinese show an SRC preference in production and (b) whether these learners’ production shows animacy effects. Overall, learners produced more target-like SRCs than ORCs, providing clear support for an SRC preference. The error patterns and individual performance also pointed toward an SRC preference. While the effect of RC type was present regardless of animacy, our findings confirmed a general animacy effect, in which learners produced more target-like RCs when these involved an animate subject and inanimate object than when subject and object were both animate. Below, we discuss the broader implications of our findings.

### Subject-Object Asymmetry in Relative Clauses

The underlying source of RC asymmetry and the NPAH is a longstanding question. There are roughly five proposals: resource-based effects, structural effects, canonicity effects, distribution-based effects, and prominence effects (Lau & Tanaka, 2021). While they all predict an SRC preference in English, they generate different predictions for Chinese RCs. We summarize them below, considering whether they are compatible with our findings.

#### Resource-Based Effects

Comprehending and producing relative clauses requires establishing and resolving a dependency between the head (filler) and the gap. A longer (linear) distance of this filler-gap dependency creates a higher cognitive load as the parser must hold and then integrate information about the filler-gap relation (e.g., Gibson, 1998, 2000). In English, the filler-gap distance is shorter in SRCs (6a) than in ORCs (6b), requiring less cognitive resources.
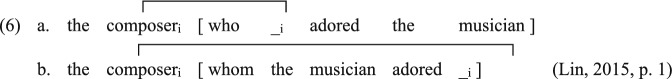


Chinese RCs are prenominal and the distance is shorter in ORCs (7b) than in SRCs (7a). Thus, ORCs require less resources than SRCs (e.g., Gibson & Wu, 2013; Hsiao & Gibson, 2003).
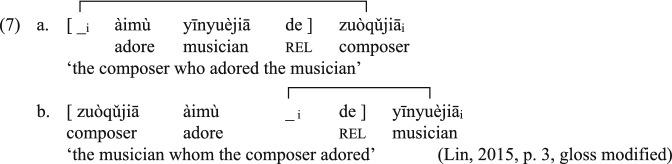


While this account is compatible with some previous findings, it is not compatible with our data.

#### Structural Effects

The source of the subject preference might lie in the structural difference between SRCs and ORCs. One way to capture the difference is to measure the structural distance between gap and head: ORCs have more XP nodes and thus are more deeply embedded than SRCs (e.g., Collins, 1994). This is true for both English and Chinese, as shown in (8).
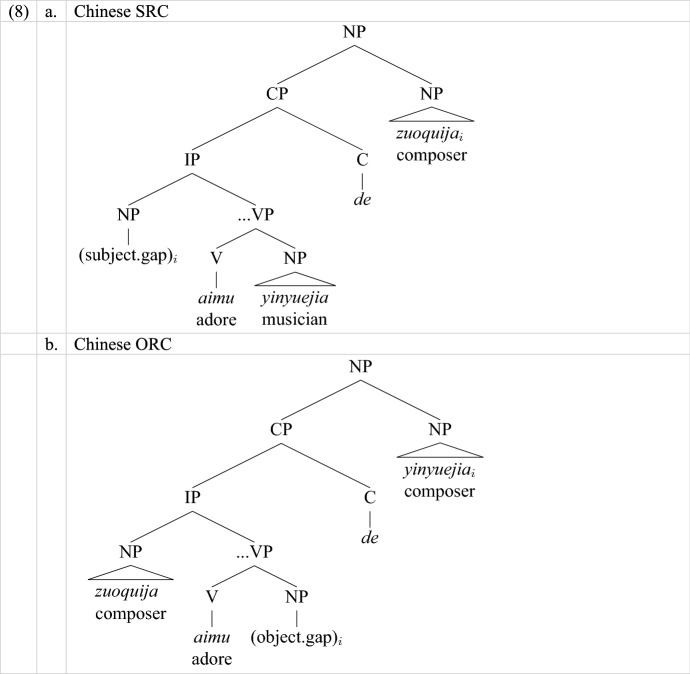


Another structural difference between SRCs and ORCs is shown in intervention effects. Building on the idea of relativized minimality (Rizzi, 1990), intervention effects are defined as the failure to establish the relation between head and gap due to an intervener’s featural similarity to the head, although the relevant features depend on language, and adults and children perceive similarity differently (e.g., Friedmann et al., 2009). Both types of structural explanations are compatible with our data.

#### Canonicity Effects

A canonical sentence schema facilitates the interpretation and production of RCs that are similar to monoclausal declaratives with canonical word order (e.g., Bever, 1970; MacDonald & Christiansen, 2002; see also Lin, 2014, 2015 for thematic ordering effects). English SRCs have SVO word order like simple transitive sentences, giving them an advantage over ORCs, which have OSV word order. In Chinese, on the other hand, ORCs (9b), but not SRCs (9a), resemble the canonical SVO word order. This account therefore predicts an ORC advantage in Chinese RCs, which is not compatible with our data.

**Table:** 

(9)	a.		V	O		S		
		[ __i_	àimù	yīnyuèjiā	de ]	zuòqǔjiā﻿_﻿i﻿_		
			adore	musician	rel	composer		
		‘the composer who adored the musician’						
	b.	S	V			O		
		[ zuòqǔjiā	àimù	﻿_﻿_i_	de ]	yīnyuèjiā_i_		
		composer	adore		rel	musician		
		‘the musician who the composer adored’					(Lin, 2015, p. 3; gloss modified)	

#### Distribution-Based Effects

An SRC preference can also be explained in terms of expectation based on relative frequency (Hale, 2001; Levy, 2008). In many languages, including Chinese, SRCs are more frequent than ORCs (Jäger et al., 2015). Without comprehensive knowledge of our participants’ input, however, we cannot ascertain whether this account is compatible with our results. There are also more nuanced distribution-based accounts that consider differences in SRCs and ORCs, such as in head animacy, a point we return to below.

#### Prominence Effects

Prominence accounts (e.g., O’Grady, 2011) would attribute the SRC preference to the semantic-pragmatic prominence of the subject, which is also the default topic of the sentence, making it easier to establish an aboutness relationship. This account predicts an SRC preference for both English and Chinese and is also compatible with our data.

### Typology of Relative Clauses

Some scholars claim that in Chinese (and other languages), what we commonly refer to as RCs are in fact attributive clauses in which the gap-head relation is not syntactic but instead constrained by semantics and pragmatics (e.g., Comrie, 1998; Matsumoto, 1990). The argument is based on the existence of “gapless RCs” in these languages (e.g., Lin, 2018; Matsumoto, 1990), such as (10) in Chinese, in which an aboutness (rather than syntactic) relationship exists between the head and the prenominal clause.

**Table:** 

(10)	[ John	chǎo	cài ]	de	wèidào
	John	stir.fry	vegetables	rel	smell
	‘the smell of John’s stir-frying vegetables’				(Lin, 2018, p. 765)

Following this argument, a subject preference would not manifest clearly due to such qualitative differences in the structure of what we generally call RCs (Ozeki & Shirai, 2007). This interpretation, however, only obtains if the source of RC asymmetry is indeed syntactic (Comrie, 2007). It is also possible that Chinese has both gapped and gapless RCs: Lin (2018) found a processing difference between genitive RCs and gapless RCs. While the underlying structure of Chinese RCs is beyond the scope of this study, our data indicate a clear subject preference in Chinese RCs, regardless of their underlying structure.

### The Role of Animacy

Our results show that there is an animacy effect that influences both SRCs and ORCs. There are two possible accounts for this result.

One account is similarity-based interference, which postulates that it is difficult to build a sentence around two similar NPs (Gordon et al., 2001). This account would predict that if there is dissimilarity in animacy between the head and the embedded NP, the RC would be easier to produce and interpret than when the animacy of the two NPs is the same (i.e., both animate or both inanimate). This account would explain why the difference in animacy improved the global performance in RC production, regardless of RC type. While there are findings against this account (e.g., MacDonald et al., 2020; Traxler et al., 2002) and relevant L2 research is scarce, Cunnings & Fujita (2021) observed similarity interference effects in the comprehension of RCs by both NSs and learners of English when the type of nominal (i.e., common nouns vs. proper nouns) was manipulated.

Another account is based on distributional frequency. SRCs often appear with animate heads and ORCs with inanimate heads: such distributional information influences acquisition, comprehension, and production, according to Kidd et al.’s (2007) account for data from English- and German-speaking children. Studies on L2 production report a strong association between SRCs and animate heads as well as between ORCs and inanimate heads based on the same logic (Jeon & Kim, 2007 for Korean; Ozeki & Shirai, 2007 for Japanese). While we do not have the distributional information available to learners, Hsiao and McDonald (2016) found a similar distribution in Chinese corpus data: the most common pattern for declarative clauses is to have an animate subject and an inanimate object, which can influence the expectation about the head animacy, and ORCs typically involve an inanimate head and an animate embedded NP. They also found that subject-modifying SRCs were more likely to appear with an animate head and an inanimate embedded NP, whereas in the object-modifying position, there was no preference as to the head animacy but inanimate embedded NPs were preferred. Hsiao and McDonald (2016) further demonstrated that animate-headed ORCs are often produced with passives by Chinese NSs. Based on these findings, it is possible that both SRCs and ORCs were easier to produce in the animate-inanimate condition than in the animate-animate condition because the former followed the most expected animacy configuration.

## Conclusion

This paper investigated whether L1-English learners of Chinese show a subject preference in the oral production of Chinese RCs and whether there is an animacy effect. While previous research on Chinese has yielded conflicted findings, our results point toward a clear subject preference, attested at both group and individual levels. Our study also corroborates the appropriateness of a picture-based oral production task to test learners’ RC knowledge avoiding comprehension-related ambiguity effects and orthography-related issues. We also observed some effect of animacy, although it did not differentially influence SRCs and ORCs, unlike what previous research suggested. Instead, RCs involving two animate NPs were generally found more difficult to produce than RCs involving an animate subject and inanimate object, indicating that global similarity-based interference or distribution-based effect may influence the difficulty of both SRCs and ORCs. We suggest this venue of inquiry for future research, along with testing performance of learners with higher proficiency and with different L1 backgrounds.

## Supplementary Information

Below is the link to the electronic supplementary material.

Supplementary file1 (DOCX 21 KB)

Supplementary file2 (PDF 89 KB)

## Footnotes

1 Among L2 studies, Chen (1999) and Xu (2013, 2014b) examined the effect of the presence and/or position of the determiner/classifier. While Jäger et al. (2015) used a determiner to eliminate the ambiguity effects, this was not the purpose of these L2 studies and it is not clear whether the determiner/classifier served as a disambiguating cue for learners.

2 A picture selection task such as the one used in Kanno (2007) would be a way to test comprehension without introducing orthography-related issues; however, it would still be subject to main clause ambiguity effects, although pictures and appropriate prompts may help reduce such effects.

3 Previous studies discussed L1 transfer in terms of different properties, but the most relevant to our study is the effect of L1 headedness and word order. When constituent orders of English and Chinese RCs are compared, Chinese ORCs and English SRCs share the SVO constituent order., but the RC head is in different places. i.e., ([SV]O) and (S[VO]), respectively. In comprehension, this might result in the misidentification of the referent, as reported in O’Grady et al.’s (2003) study involving L1-English learners of Korean. In production, Yip & Matthews (2007) found transfer of pre-nominal modification strategy from Cantonese to English in Cantonese-English bilingual children. While we cannot draw a conclusion about transfer effects without another L1 group, some of the utterances produced seem to suggest that L1-English learners transferred English relativization strategy to Chinese. We come back to this point in footnote 6.

4 Participants’ proficiency was measured using an independent proficiency measure reported in the section on Picture Narration Task. We did not measure their proficiency using a standard scale, but the Chinese program we recruited participants from set the target level on the American Council on the Teaching of Foreign Languages (ACTFL) Oral Proficiency Interview (OPI) scale of novice-high/intermediate low for students completing the first year, intermediate-mid for students completing the second year, intermediate-high for students completing the third year. This means our participants’ proficiency level ranged roughly between intermediate-low and intermediate-high levels on the ACTFL scale.

5 Blocked design is often used for child language research because constantly switching A–A and A–I items might be tasking (e.g., Chan et al., 2009); we decided to implement the same safeguard for learners. Moreover, the A–I items and A–A items used different sets of verbs, making the differences (visually and linguistically) between the two animacy conditions greater than the differences between the two RC conditions. Our main comparison of interest was between SRCs and ORCs, and the blocked design helped us make this comparison more directly while maintaining the other factors constant.

6 Among 213 responses (95 in SRC, 118 in ORC) categorized as “failure to produce RC,” there were 34 responses (9 in SRC, 25 in ORC) considered acceptable as they identified the correct referent but did not feature an RC (e.g., genitives, comitatives, etc.). Six responses (all in ORC) included only the head NP; 154 responses (77 in SRC, 77 in ORC) were simple declaratives describing the picture, which are not pragmatically felicitous given the question ‘Who/What is the arrow pointing to?’. In addition, 19 responses (9 in SRC, 10 in ORC) consisted of a pattern in which a declarative was introduced by the copula *shì*, occasionally preceded by a pronoun or a phrase. For example, (i) was produced instead of the target RC “The girl who is kicking the female nurse.”

**Table:**

(i)	Zhè	shì	nǚhái	tī	nǚ-hùshì
	This	COP	girl	kick	female-nurse

This is possible evidence of L1 transfer: participants tried to produce a reduced RC, such as ‘(This) is the girl kicking the female nurse,’ thus inserting a copula followed by a postnominal RC (without a relativizer). This resembles the transfer of Cantonese prenominal modification strategy to English, observed by Yip & Matthews (2007) in Cantonese-English bilingual children. Alternatively, this sentence pattern could represent a type of cleft, called bare-*shì* (Paul & Whitman, 2008), which learners were not taught but may have encountered outside of classroom. Moreover, since participants had been instructed on standard *shi…de* clefts at the same time as RCs, they may have attempted to produce a cleft such as ‘It is the girl that kicks the female nurse’ in lieu of an RC. Clefts, however, cannot be preceded by a pronoun or a phrase, unlike what we observed in our data (*cf*. (i)), and they are not the most pragmatically appropriate responses given the the context, as indicated by the fact that NSs produced none. Whether this pattern is due to L1 transfer or not needs to be confirmed in a future study involving another L1 group.

7 We suspect that this may be due to some type of learning effect, i.e., starting from a more difficult condition helped perform better in an easier condition.

8 Volpato and Adani (2009) tested comprehension and their response options were binary. For our production task, chance criterion could be lowered to .25 (*p* = 0.02) and .20 (*p* = 0.03) based on four response categories for SRCs and five for ORCs (Table 2), increasing the number of “successful with both” learners to 17 and reducing the number of “successful with neither” ones to just six. Yet the subject preference would still hold, with seven learners being more successful with SRCs, and none being more successful with ORCs.

## Declarations

### Availability of Data and Material

Data supporting the finding of this study are not openly available because participants of this study did not agree for their data to be shared publicly. Derived data supporting the findings of this study are available from the corresponding author on reasonable request.
