# Supplementary material for: Subject Advantage in L1-English Learners’ Production of Chinese Relative Clauses
Source: J Psycholinguist Res. 2022 Apr 24;52(2):405–24. doi: 10.1007/s10936-022-09865-9 (PMC10163100; doi:10.1007/s10936-022-09865-9)
Supplement: Supplementary file 1 — Supplementary file1 (DOCX 21 KB) [file 10936_2022_9865_MOESM1_ESM.docx]

**Supplementary File 1: Learners’ Demographic Information, Proficiency Scores, and Targetlike RC Tokens**

| Participant | Gender | Age of testing | Age of L2 onset | Proficiency | | | | RC production | |
| --- | --- | --- | --- | --- | --- | --- | --- | --- | --- |
|  |  |  |  | Verbal density (raw score) | Lexical complexity (raw score) | Accuracy (raw score) | Combined *z*‑score | SRC | ORC |
| 2Y1 | F | 19 | 17 | 1.71 | 0.86 | 0.52 | −0.73 | 0 | 4 |
| 2Y2 | M | 18 | 17 | 1.55 | 0.91 | 0.74 | 1.73 | 7 | 1 |
| 2Y3 | F | 18 | 17 | 1.88 | 0.92 | 0.35 | 0.52 | 3 | 3 |
| 2Y4 | M | 18 | 16 | 2.25 | 0.83 | 0.56 | 0.37 | 10 | 9 |
| 2Y5 | M | 20 | 19 | 1.67 | 0.87 | 0.28 | −2.07 | 7 | 4 |
| 2Y6 | F | 19 | 18 | 1.91 | 0.85 | 0.41 | −0.97 | 0 | 3 |
| 2Y8 | M | 19 | 18 | 1.47 | 0.85 | 0.40 | −2.47 | 9 | 6 |
| 2Y9 | M | 19 | 18 | 1.21 | 0.86 | 0.71 | −0.95 | 8 | 6 |
| 2Y11 | M | 19 | 18 | 1.63 | 0.89 | 0.47 | −0.40 | 8 | 8 |
| 2Y14 | M | 19 | 18 | 1.65 | 0.79 | 0.35 | −3.88 | 9 | 6 |
| 3Y1 | F | 20 | 18 | 2.08 | 0.88 | 0.75 | 2.43 | 6 | 7 |
| 3Y2 | F | 19 | 18 | 1.71 | 0.87 | 0.57 | −0.01 | 3 | 4 |
| 3Y3 | M | 20 | 15 | 1.91 | 0.89 | 0.63 | 1.50 | 6 | 5 |
| 3Y4 | M | 21 | 19 | 2.02 | 0.91 | 0.60 | 2.07 | 9 | 9 |
| 3Y5 | M | 21 | 18 | 1.47 | 0.94 | 0.58 | 1.16 | 9 | 8 |
| 3Y6 | M | 42 | 41 | 1.76 | 0.89 | 0.52 | 0.30 | 10 | 4 |
| 3Y7 | M | 21 | 20 | 2.75 | 0.90 | 0.25 | 1.97 | 7 | 5 |
| 3Y8 | M | 20 | 18 | 1.48 | 0.90 | 0.72 | 1.00 | 6 | 5 |
| 3Y9 | F | 20 | 16 | 1.62 | 0.90 | 0.62 | 0.66 | 1 | 0 |
| 3Y10 | F | 19 | 16 | 1.74 | 0.86 | 0.53 | −0.63 | 8 | 2 |
| 3Y11 | M | 20 | 18 | 1.64 | 0.89 | 0.36 | −1.19 | 9 | 6 |
| 3Y12 | M | 21 | 18 | 2.08 | 0.79 | 0.33 | −2.78 | 1 | 1 |
| 3Y14 | M | 60 | 57 | 2.00 | 0.86 | 0.25 | −1.57 | 4 | 1 |
| 3Y15 | F | 22 | 18 | 1.27 | 0.89 | 0.67 | −0.17 | 9 | 2 |
| 3Y16 | F | 19 | 14 | 1.91 | 0.92 | 0.59 | 2.00 | 8 | 9 |
| 3Y17 | M | 19 | 18 | 1.52 | 0.88 | 0.52 | −0.76 | 10 | 7 |
| 3Y19 | M | 19 | 15 | 1.44 | 0.90 | 0.47 | −0.66 | 5 | 0 |
| 3Y20 | F | 20 | 15 | 1.25 | 0.88 | 0.63 | −0.77 | 6 | 9 |
| 3Y21 | M | 21 | 18 | 1.85 | 0.94 | 0.77 | 3.54 | 10 | 10 |
| 3Y22 | F | 22 | 15 | 1.56 | 0.89 | 0.67 | 0.76 | 10 | 9 |

*Note.* The first two letters of the participant code represent instructional level: 2Y = 2nd year, 3Y = 3rd year.
