# Supplementary material for: Subject Advantage in L1-English Learners’ Production of Chinese Relative Clauses
Source: J Psycholinguist Res. 2022 Apr 24;52(2):405–24. doi: 10.1007/s10936-022-09865-9 (PMC10163100; doi:10.1007/s10936-022-09865-9)
Supplement: Supplementary file 2 — Supplementary file2 (PDF 89 KB) [file 10936_2022_9865_MOESM2_ESM.pdf]

Supplementary File 2: List of Test Sentences

1. Animate-animate, subject relative clauses

**Prompts**	**English translation**	**Target response**	**English translation**
这个女孩在打女医生。这个女孩在打男医生。箭头指着谁?	This girl is hitting a female doctor. This girl is hitting a male doctor. Who is the arrow pointing to?	在打男医生的女孩	The girl who is hitting the male doctor
这个女孩在踢男护士。这个女孩在踢女护士。箭头指着谁?	This girl is kicking a male nurse. This girl is kicking a female nurse. Who is the arrow pointing to?	在踢女护士的女孩	The girl who is kicking the female nurse
这个儿子在想妈妈。这个儿子在想爸爸。箭头指着谁?	This son is missing his mother. This son is missing his father. Who is the arrow pointing to?	在想妈妈的儿子	The son who is missing his mother
这个男老师在教男学生。这个男老师在教女学生。箭头指着谁?	This male teacher is teaching a male student. This male teacher is teaching a female student. Who is the arrow pointing to?	在教女学生的男老师	The male teacher who is teaching the female student
这个男孩在等男服务员。这个男孩在等女服务员。箭头指着谁?	This boy is waiting for the male server. This boy is waiting for the female server. Who is the arrow pointing to?	在等男服务员的男孩	The boy who is waiting for the male server

2. Animate-animate, object relative clauses

**Prompts**	**English translation**	**Target response**	**English translation**
男孩在打这个男医生。女孩在打这个男医生。箭头指着谁?	The boy is hitting this male doctor. The girl is hitting this male doctor. Who is the arrow pointing to?	男孩在打的男医生	The male doctor whom the boy is hitting
男孩在踢这个男护士。女孩在踢这个男护士。箭头指着谁?	The boy is kicking this male nurse. The girl is kicking this male nurse. Who is the arrow pointing to?	男孩在踢的男护士	The male nurse whom the boy is kicking
女儿在想这个爸爸。儿子在想这个爸爸。箭头指着谁?	The daughter is missing her father. The son is missing his father. Who is the arrow pointing to?	女儿在想的爸爸	The father whom the daughter is missing
女老师在教这个女学生。男老师在教这个女学生。箭头指着谁?	The female teacher is teaching this female student. The male teacher is teaching this female student. Who is the arrow pointing to?	男老师在教的女学生	The female student whom the male teacher is teaching
男孩在等这个女服务员。女孩在等这个女服务员。箭头指着谁?	The boy is waiting for this female server. The girl is waiting for this female server. Who is the arrow pointing to?	男孩在等的女服务员	The female server whom the boy is waiting for

3. Animate-inanimate, subject relative clauses

Prompts	English translation	Target response	English translation
这个女孩在喝可乐。这个女孩在喝查。箭头指着谁？	This girl is drinking cola. This girl is drinking tea. Who is the arrow pointing to?	在喝可乐的女孩	The girl who is drinking cola
这个女孩在吃汉堡包。这个女孩在吃糖醋鱼。箭头指着谁？	This girl is eating a hamburger. This girl is eating sweet and sour fish. Who is the arrow pointing to?	在吃汉堡包的女孩	The girl who is eating the hamburger
这个男孩在做饺子。这个男孩在做蛋糕。箭头指着谁？	This boy is making dumplings. This boy is making a cake. Who is the arrow pointing to?	在做蛋糕的男孩	The boy who is making the cake
这个女孩在用手机。这个女孩在用电脑。箭头指着谁？	This girl is using the cellphone. This girl is using the computer. Who is the arrow pointing to?	在用手机的女孩	The girl who is using the cellphone
这个男孩在洗苹果。这个男孩在洗杯子。箭头指着谁？	This boy is washing an apple. This boy is washing a cup. Who is the arrow pointing to?	在洗杯子的男孩	The boy who is washing the cup

4. Animate-inanimate, object relative clauses

Prompts	English translation	Target response	English translation
女孩在吃这个汉堡包。男孩在吃这个汉堡包。箭头指着什么？	The girl is eating this hamburger. The boy is eating this hamburger. What is the arrow pointing to?	男孩吃的汉堡包	The hamburger that the boy is eating
男孩在喝这个可乐。女孩在喝这个可乐。箭头指着什么？	The boy is drinking this cola. The girl is drinking this cola. What is the arrow pointing to?	男孩在喝的可乐	The cola that the boy is drinking
女孩在做这个蛋糕。男孩在做这个蛋糕。箭头指着什么？	The girl is making this cake. The boy is making this cake. What is the arrow pointing to?	男孩在做的蛋糕	The cake that the boy is making
女孩在用这个手机。男孩在用这个手机。箭头指着什么？	The girl is using this cellphone. The boy is using this cellphone. What is the arrow pointing to?	女孩在用的手机	The cellphone that the girl is using
女孩在洗这个苹果。男孩在洗这个苹果。箭头指着什么？	The girl is washing this apple. The boy is washing this apple. What is the arrow pointing to?	女孩在洗的苹果	The apple that the girl is washing
